# The post-conflict expansion of coca farming and illicit cattle ranching in Colombia

**DOI:** 10.1038/s41598-023-28918-0

**Published:** 2023-02-03

**Authors:** Paulo J. Murillo-Sandoval, John Kilbride, Elizabeth Tellman, David Wrathall, Jamon Van Den Hoek, Robert E. Kennedy

**Affiliations:** 1 College of Earth, Ocean and Atmospheric Sciences, Oregon State University, Corvallis, OR USA; 2 Departamento de Topografía, Facultad de Ciencias del Hábitat, Diseño e Infraestructura, Universidad del Tolima, Ibagué, Colombia; 3 School of Geography, Development, and Environment, University of Arizona, Tucson, AZ USA

**Keywords:** Environmental sciences, Environmental social sciences

## Abstract

Illicit cattle ranching and coca farming have serious negative consequences on the Colombian Amazon’s land systems. The underlying causes of these land activities include historical processes of colonization, armed conflict, and narco-trafficking. We aim to examine how illicit cattle ranching and coca farming are driving forest cover change over the last 34 years (1985–2019). To achieve this aim, we combine two pixel-based approaches to differentiate between coca farming and cattle ranching using hypothetical observed patterns of illicit activities and a deep learning algorithm. We found evidence that cattle ranching, not coca, is the main driver of forest loss outside the legal agricultural frontier. There is evidence of a recent, explosive conversion of forests to cattle ranching outside the agricultural frontier and within protected areas since the negotiation phase of the peace agreement. In contrast, coca is remarkably persistent, suggesting that crop substitution programs have been ineffective at stopping the expansion of coca farming deeper into protected areas. Countering common narratives, we found very little evidence that coca farming precedes cattle ranching. The spatiotemporal dynamics of the expansion of illicit land uses reflect the cumulative outcome of agrarian policies, Colombia’s War on Drugs, and the 2016 peace accord. Our study enables the differentiation of illicit land activities, which can be transferred to other regions where these activities have been documented but poorly distinguished spatiotemporally. We provide an applied framework that could be used elsewhere to disentangle other illicit land uses, track their causes, and develop management options for forested land systems and people who depend on them.

## Introduction

More than 40% of deforestation worldwide is estimated to be tied to illegal activities^1^, linked to illegal clearing for commercial agriculture and agro-commodities such as beef^2^, tropical timber^3^, oil palm^4,5^, soy^6,7^, as well as cultivation of illicit crops such as coca^8^. Earth Observation technologies offer the capacity to monitor land-use changes associated with these consequential activities. Still, it is difficult to clearly identify and characterize specific land-use changes as “illicit.” For instance, people engaged in illicit activities intentionally work to obscure traces of their behavior. Subsequently, illicit activities are not well documented in publicly accessible data, making it difficult to connect them with land cover changes observable from remote sensing^9–11^. Illicit activities may exhibit landscape patterns similar to those of legitimate activities, making it difficult to detect and differentiate illicit activity from other land-use types^12^. While causal relationships between illicit activities and land-use change at broad spatial scales have been documented (e.g., for municipalities, departments)^11,13^ illicit activities often produce localized, clustered, and context-dependent patterns. Although practical remote sensing applications have facilitated the implementation of forest conservation policies^14^, the development of analytical frameworks that identify distinct illicit land activities remains nascent in land system science. This limits the utility of many remote sensing applications for decision-makers^15–17^. Here, we apply deep learning algorithms to satellite imagery at 30 m resolution (1985–2019) to differentiate annual patterns of coca farming and cattle ranching, allowing us to link land use changes to illicit activities. We analyze trajectories of illicit land-use change caused by policy changes in Colombia regarding agrarian policies, the war on drugs, and the 2016 peace accord.

A complex interplay among poor land access regimes linked to weak institutions, narcotrafficking, and armed conflict has shaped socio-ecological conditions in Colombia. Land access and landscape changes in Colombia result from historical waves of spontaneous and state-sponsored colonization by landless farmers in peripheral lands^18^. As farmers colonized these lands, disputes with large landholders increased, and rebel groups emerged to engage in armed conflict. The conflict has determined how the land has been defined, occupied, and utilized^19^ under the umbrella of high economic returns from narcotrafficking and no other farming alternatives; the coca surplus fed the conflict over decades. During the conflict, rebel groups created specific environmental rules that shaped the landscape, such as limits on the number of clear-cuts within farms per year, constraints on agricultural expansion, and fees for clearing forests in disallowed locations^20^. Governmental policies emerged to promote the transition from coca farming to the production of livestock ranching activity as a “licit” alternative to coca^21^. However, cattle promotion is not only a commercial business but served as a strategy for the consolidation of “*baldios*” or empty forestlands by large landholders, encouraging forest clearing also inside Protected Areas (PAs). While beef consumption in Colombia is stable and has no relation with the exponential land pasture increase, people opt for cattle ranching to claim land titles and sell cattle during economic distress^22^. Weak institutions and unbalanced power structures that benefit large landholders with the capital to invest in clearing lands have characterized socio-ecological settings in Colombia. As a result, the main drivers of deforestation in the Amazon are coca farming and cattle ranching^23^.

Coca farming is the largest illicit agribusiness in the world, and Colombia is the world's leading coca producer^24^. By 2000, 60–80,000 Colombia's *cocalero* families supplied two-thirds of the world's coca and its end-product, cocaine^25^. In 2017, Colombia had 170,000 ha under coca cultivation^26^ with 47% of cultivation within areas designated for special management such as protected areas, areas occupied by indigenous groups, Afro-descendent territories, and forest reserve areas^26^. Coca farming is not a dominant driver of deforestation at the national level. However, it is locally important and can be embedded in the agricultural frontier associated with large-scale agricultural projects^13^. In Colombia, coca farming has helped shape the agricultural frontier, defined in 2018 as the boundary separating lands where agricultural activities are allowed from protected areas, areas of special ecological importance, and other areas where agricultural activities are otherwise excluded by law^27^. Along with coca farming, illegal cattle ranching has been a significant threat to forest conservation in the agricultural frontier, where the conversion of forest to cattle ranching is a major factor contributing to deforestation in the Amazon region^23,28^. In Colombia, forest conversion to cattle ranching serves as a mechanism for legalizing informal or illegal landholdings^21^. Farmers invest in cattle to secure claims to land speculatively connected to laundering drug profits^8,29^. Small farmers see cattle ranching as a safe investment that provides a quick return or as a buffer during economic uncertainty^22^. During recent decades of the Colombian conflict (1984–2011) between the Government of Colombia (GoC) and Fuerzas Armadas Revolucionarias de Colombia-Ejercito del Pueblo (FARC-EP), illegal cattle ranching remained low^30^. Since 2012, armed conflict intensity has declined with a significant reduction in fatalities. Still, a rapid expansion of cattle lands has been widely documented^31^, mainly linked to the peace agreement realization in 2016^32^. The agreement indicated the end of FARC-led gunpoint conservation. It signaled the beginning of new land use activities such as cattle ranching, promoted by drug cartels and large landowners seeking to capitalize on more favorable land policies^31^. A key challenge to understanding drivers of deforestation is robustly mapping the conversion between *forests, coca farms and cattle ranches.* Persistent coca farming may indicate that farmers are responding to high economic incentives from coca cultivation or that coca control measures in specific locations are ineffective. In contrast, rapid cattle expansion may support claims about the presence of illegal, speculative land markets in agricultural frontiers. While detecting these conversion patterns and identifying drivers helps to prioritize conservation efforts, distinguishing between conversion types (e.g., *forest-to-coca* and *forest-to-cattle)* is difficult because both land classifications follow similar temporal and spectral trends in remotely sensed imagery. Previous research has relied on classified or partial observations of these land uses, i.e. aerial photographs^9^. Using IKONOS imagery (1 m pixel size), Pesaresi^33^ discriminated coca from bare soil, forest, and shadows using textural metrics, but cattle ranching was absent in that study. Currently, coca discrimination from other land uses is achieved through the Colombian government's visual interpretation of aerial imagery through the Integrated System for Illicit Crop Monitoring (SIMCI). Omission errors have been reported by SIMCI caused by human interpretation in coca detection and by persistent cloud cover^34^, as well as discrepancies with other data sources such as the Office of National Drug Control Policy^35^. Consequently, identifying illicit cattle ranching and coca cultivation with available remote sensing imagery remains a significant challenge across Latin America^9,36^.

To link illicit coca farming and cattle ranching activities to observable land change patterns in the Colombian Amazon, we overcome previous challenges with two complementary approaches. The first approach hypothesizes that unique, observable patterns can be linked to known historical and institutional processes that drive them (specifically the peace negotiation process for the Colombian civil war and significant anti-coca policies employed during Colombia's War on Drugs). The second approach leverages known observations of illicit activities to classify pixel patterns or objects that exhibit statistically similar variables using a deep learning (DL) algorithm. While both approaches have been used separately in remote sensing^37^, we show they can synergistically identify specific landscape patterns associated with illicit land activities^12^. Together, these two approaches explain and quantify specific *forest-coca-cattle* change patterns and their associated drivers (e.g., historical agrarian policies). To evaluate the role of illicit land activities on forest change, we answer two questions:

1. Do illicit activities (cattle ranching and coca farming) drive distinct, evolving land change patterns in the Colombian Amazon?
2. How do policy regimes in the Colombian Amazon, such as agrarian policies, the War on Drugs (i.e., coca substitution and aerial fumigation campaigns), and the recent peace agreement, relate to the expansion of illicit land activities?

## Tracking illicit land activities in the Amazon

**Figure 1:**
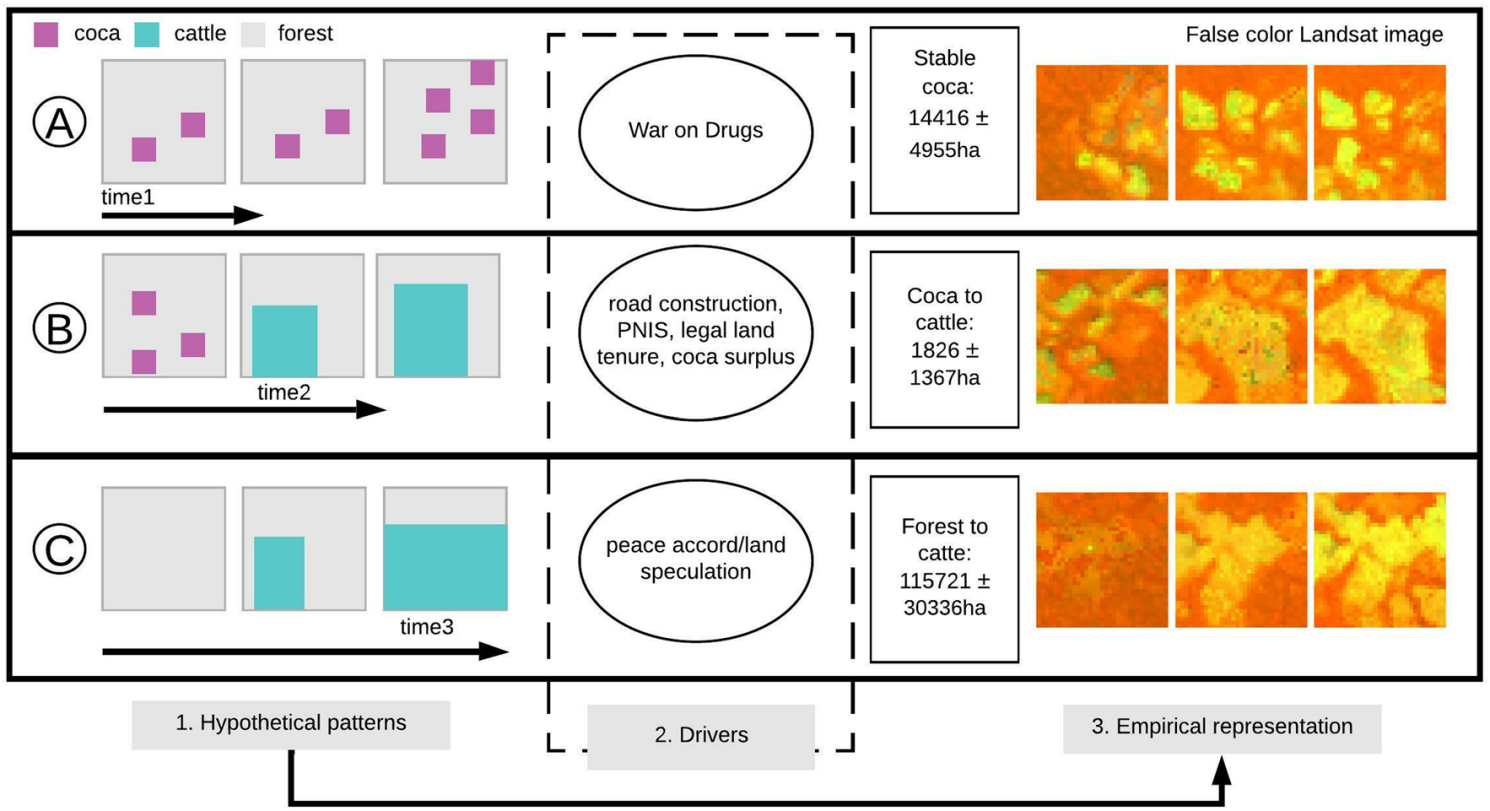
A conceptual framework describing hypothetical and empirical land change patterns arising from different drivers of illicit land use activities. (1) Hypothetical patterns depend on historical institutional processes: (**A**) The War on Drugs and perverse incentives for coca substitution promote coca stabilization within PAs; (**B**) agrarian policies, such as the Integrated National Program for the Substitution of Illicitly Used Crops (PNIS), that promote cattle as a strategy less "illicit" than coca farming promote conversion from coca to cattle; (**C**) peace accord policies that enhanced land speculation for cattle ranching. (2) While drivers are not mutually exclusive, places outside the agricultural frontier (PAs and deeper regions of the Amazon) provide incentives for illicit activities that lead to consolidating coca and cattle to obtain rent and guaranteed economic returns for small farmers. (3) Coca and cattle lands produce diverse land-use patterns in the observed Landsat time-series (NIR, SWIR1, Red—false color combination). The areas reported refer to the mean and standard deviation area of change during the 34-year study period in the Amazon.

Our study uses two pixel-based approaches to understand how illicit land activities accelerate land-use change. Figure 1 depicts a conceptual framework of process-based insights on hypothetical linkages between human-induced illicit activities and observed pixel patterns^12^. We summarized the role of illicit activities using the three most frequently documented *forest-coca-cattle* transition pathways A) stable coca, B) coca to cattle, and C) forest to cattle (Fig. 1). Each of these pathways would leave a hypothetical pattern (Fig. 1(1)), which could be detected empirically (Fig. 1(3)). This first approach is needed given the absence of sufficient on-the-ground records of coca and cattle that would typically be required to classify remote sensing data directly.

Hypothesized spatiotemporal forest, coca, and cattle conversion pathways and their drivers in Colombia are depicted in Fig. 1. *Stable coca* farming can be linked to agrarian policies that have promoted state-sponsored mass migration since 1953^18,38^. While these policies failed to promote effective legal incentives for agriculture, more profitable alternatives than traditional agriculture resulted in booms of marijuana production in 1974, followed by coca in 1978^39^. These booms caused the expansion of small coca patches in clustered locations (Fig. 1A). Coca is persistent because it is a cash economic alternative for farmers and also because the War on Drug policies created a "balloon effect"^40,41^ in which coca moved within ecologically sensitive zones (e.g. PAs) to avoid aerial fumigation. This enabled FARC-EP institutional control over coca production, allowing FARC-EP to tax coca growers and merchants^18,39^ and thus finance war-fighting capabilities^42^. Because the coca economy funded the armed conflict, the GoC and small farmers developed coca substitution programs (i.e., PNIS—Programa Nacional Integral de Sustitución de Cultivos de Uso Ilícito) to enroll farmers into the legal economy^43^. However, PNIS faces significant setbacks^44^. Coca substitution for other crops within PAs is constitutionally impossible^45^, technical assistance is delayed^46^, and many PNIS leaders have been killed^47^. Uncertainty about the PNIS and the longevity of legal guarantees to support families specifically within PAs contribute to the persistence of coca farming or the transition to cattle ranching.

*Coca to cattle* results from the progressive substitution of coca production with less "illicit" alternative activities^21,48^. Three possible causes of this transition have been noted. First, the expansion of the road network by FARC-EP command (~ 200 km)^49^ led to an increase in commercializing land, and motivated actors to expand forest clearing^49,50^. Second, agrarian policies that incentivized legal ownership were conditioned on 'land productivity', most efficiently demonstrated through cattle establishment^21^. Third, surplus profits from coca production allowed farmers to acquire and gradually merge abandoned coca plots, eventually converting them into pasture^29,51^. This conversion is carried out by farmers who allow the transit of cattle from other non-state actors in their farms, returning economic incentives to both groups^29,38^. This spatial pattern has been gradual and more evident after 2008^30,52^, and has led to large cattle farms being surrounded by remaining small coca plots (Fig. 1B).

Massive *forest to cattle* conversion is not driven by small farmers^2^. Rather, it is more often driven by non-state actors who have capital to invest in forest clearing and who may be connected with coca profits^8,53^. This process has been documented after the peace accord, when the vacuum produced by de facto FARC-EP demobilization enabled the incursion of actors interested in expanding cattle ranching. This conversion pattern is presented within PAs borders and deeper in the Amazon, with mean deforested patch size greater than 7ha^31^ (Fig. 1C). While decreased coca cultivation coincides with reduced cattle ranching expansion^29,38^, the acute massive conversion of *forest to cattle* since 2017 may be linked to external land speculators –investors–who see future opportunities to capitalize on land.

The second approach employs partially available records of coca and cattle to directly link remote sensing data (empirical) with illicit land activities. Coca and illicit cattle ranching have distinct spatial patterns which can be captured by convolutional DL algorithms with greater accuracy than conventional pixel-by-pixel or object-based classification methods^54^. While satellite representation indicates that distinguishing between coca and cattle is very challenging, the capacity for pattern recognition in spatial, spectral, and temporal dimensions of the image makes DL algorithms effective for classifying common and recurring patterns^55^. Leveraging these two-based pixel approaches, we overcome the difficulty of studying the real impact of illicit land activities on the landscape. Synergies between both approaches also support the creation of specific spatiotemporal linkages between land uses that are lacking in land system science. Moreover, it opens new opportunities to examine the role of other illicit activities on global environmental change.

## Results

**Figure 2:**
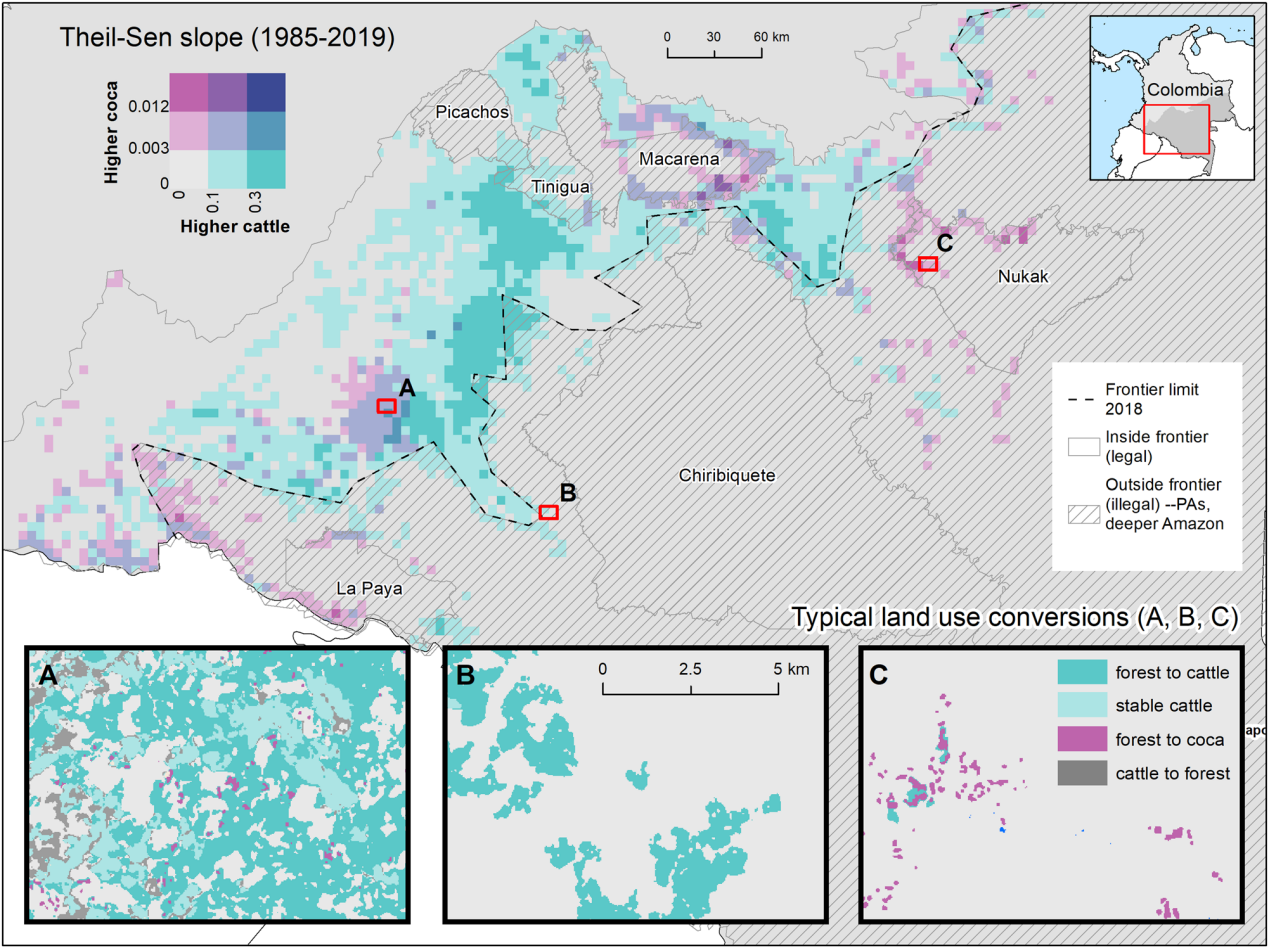
Trends in land conversion in the Colombian Amazon. Mapped area of coca and cattle was aggregated to 5 km cells, and trends in area were summarized using the Theil Sen slope calculation. (**A**) Region with transition from coca to new large cattle lands. (**B**) Large-scale illegal conversion past the agricultural frontier, deeper into the Amazon. (**C**) Coca plots expansion within the Nukak Makú indigenous reserve. Map created in QuantumGIS 3.16 (https://www.qgis.org/en/site/).

**Figure 3:**
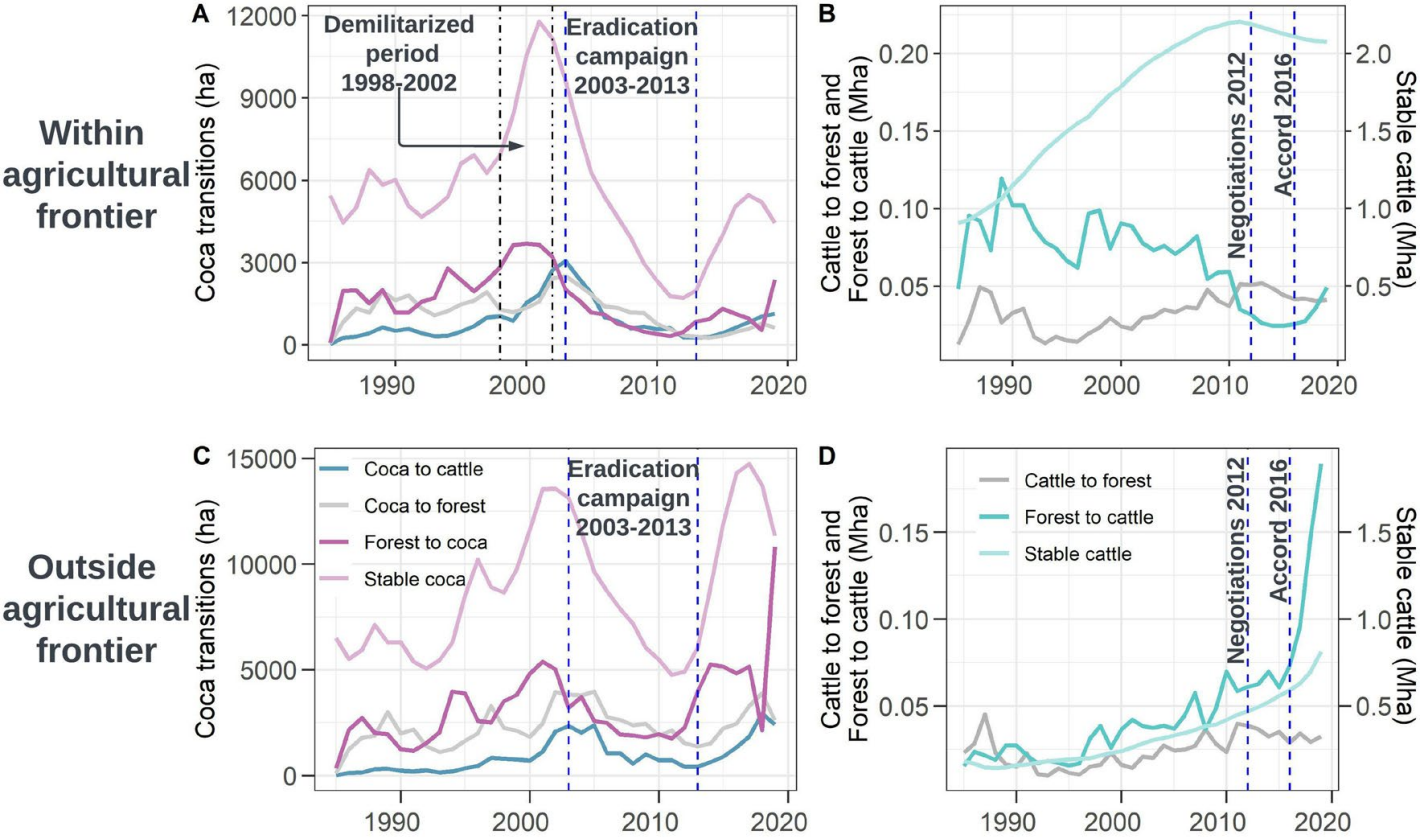
Mapped areas for the conversion patterns *forest-coca-cattle*. First row represents conversion patterns within the legal agricultural frontier defined in 2018, second row shows the patterns outside the frontier, in which agriculture is considered illegal.

The vast majority of deforestation (~ 3Mha) is attributed to stable cattle ranching lands, whereas coca farming accounted for 1/60th of that area –less than ~ 45,000 ha (Fig. 3). We summarized the results based on the agricultural frontier defined by the GoC in 2018. This frontier represents the boundary between areas in which agricultural activities are considered legal (the northwestern area in Fig. 2, as well as Fig. 3A,B) and where activities are illegal, such as PAs and regions deep in the Amazon (the hatched southeastern area in Fig. 2, as well as Fig. 3C,D). The *forest to cattle* conversion pattern was especially pronounced outside the agricultural frontier (Fig. 3D). Meanwhile, *stable coca* farming contributed to ~ 35.000 ha of deforestation around 2003, but with greater variation during conflict and eradication campaigns (Fig. 3A,C). The conversion of *coca to cattle* represents only a small fraction of total land use change within the agricultural frontier, outside it, and within PAs (< 4000 ha), sharply challenging popular narratives that cattle ranching is a common strategy for legitimizing coca plots. The performance of the DL model using the mean Intersection over Union (mIoU) was 0.62 (± 0.04), 0.92 (± 0.014), 0.92 (± 0.013) for coca, cattle, and forest areas respectively. A fully independent accuracy assessment for the nine map change classes shows a global accuracy of 0.96 (± 0.04). Specific details for area estimation and confidence intervals for each change class are included in the SI.

The scale of* forest to cattle* is unprecedented. This dramatic transition occurred outside of the agricultural frontier following the peace accord. Although deforestation was expected after the accord^19,56^, the magnitude of this outbreak has no precedent in our 34-year study. It illustrates a new cattle regime driven by speculative illegal markets in which different actors have contributed to the massive clearing of forested lands^31^. While *forest to cattle* conversion following the negotiation process was muted within the established agricultural frontier (Fig. 3B), we detected a peak of remaining forest loss between 2016 and 2019. Within these three years, illegal *stable cattle* rose by ~ 800,000 ha outside the frontier (Fig. 3D), roughly half the area of *stable cattle* within the official frontier (~ 2 Mha) (Fig. 3B). Since at least the early 1990s, *stable cattle* has been progressively consolidating outside the frontier, though the rate of expansion has greatly accelerated since the accord was signed (Fig. 3D).

*Stable coca* in 2018 is roughly 2.5 times more abundant outside the agricultural frontier than inside (Fig. 3A,C). It also presented a spike with new cultivation increasing after the eradication campaign from ~ 50,000 ha in 2013 to ~ 15,000 ha in 2018 (Fig. 3C). The conversion of *forest to coca* within the agricultural frontier was much more limited (~ 5500 ha, Fig. 3A). While the prevalence of coca outside the frontier reflects the imperative of coca farmers to obscure their activities from authorities, coca has remained largely concentrated in the same hotspots during the study period (Fig. 2). Coca increases outside the frontier also coincide with the timing of coca booms, when coca prices increased in 1984–1990^18^ and 1998–2002^29^, which was also when FARC-EP was legally settled in the region. The decrease in coca inside and outside the agricultural frontier coincided with eradication campaigns (2003–2013), when coca decreased to its minimum levels until < 3000 ha in 2010 (Fig. 3A). As the peace process advanced, beginning in about 2012, *forest to coca* conversions increased again, following a similar pattern of expanding coca cultivation that occurred during demilitarization period 1998 to 2002.

Findings of smaller coca transitions run counter to the dominant popular narrative that the conversion from *coca to cattle* is a relevant transition in the Amazon (Fig. 3A,C). *Coca to cattle* follows a similar trend of *stable coca* within the frontier (Fig. 3A), but outside the frontier *coca to cattle* is less common than *coca to forest* and f*orest to coca* conversions (Fig. 3C). These results suggest that coca farming is a preferable option within and outside the frontier, and also that coca is more commonly abandoned (i.e., leading to forest regrowth) rather than converted to cattle lands. Despite the overwhelming influence of cattle ranching outside the agricultural frontier, the average annual area of coca to cattle conversion is ~ 1000 ha, with a maximum value of 3000 ha in 2003 (Fig. 3C), representing the smallest conversion category. Interestingly, the *cattle to forest* conversion leading to secondary forest (Fig. 3B,D) is more prominent than other kinds of coca transition (Fig. 3A,C). While this critical conversion was not included in our hypothesized patterns (Fig. 1), the gradual increase from 2000 to 2012 indicates the resilience of the Amazon forest ecosystem^57^.

Protected Areas remain havens for both coca and new cattle consolidation lands, which are most heavily concentrated in the Protected Areas of Macarena, Nukak, La Paya, and Tinigua (Fig. 2, see SI). The direct conversion from *forest to cattle* has irreversible consequences for pristine forests in Tinigua and Macarena (SI Fig. S5C, S5F). Patterns of *forest to cattle* in Tinigua show how old and new farmers have purposefully fragmented and appropriated Tinigua with sponsorship by FARC-EP dissidents and larger ranchers^58,59^. Dissidents have territorial control in Guaviare and Putumayo, encouraging coca production^60,61^ In Macarena, *forest to cattle* conversion has been slower, confined mostly to the park's borders and not in the core area. Coca farming is most abundant in Macarena, where it has been persistent since 1985 and has moved ~ 5 km inside PA boundaries (see SI Fig. S5A, S5D). The Nukak Makú Indigenous reserve has the second largest area affected by coca farming (see Fig. S5A, S5D); this alarming increase puts Indigenous peoples at risk of conflict with outsiders and government interdiction forces. As with all other areas, in both PA buffer zones and in their core areas, *forest to coca* conversions prevail over *coca to cattle* conversion (see Fig. S5B, S5E). All mapped area transitions within and outside the agricultural frontier are included in the SI.

## Discussion

We find illicit activities (cattle ranching and coca farming) drive diverse land change patterns during the last 34 years. Using DL algorithms, we confirm the presence of hypothesized conversion patterns between *forest-coca-cattle,* and we disentangle heretofore indistinguishable land use changes and their trajectories by exploiting the spatial context in optical imagery. Our findings show that under the high uncertainty of the peace negotiation process, farmers prioritized coca farming. This is also linked to the long period when the GoC failed to create real alternatives to coca farmers in the study area^62,63^. In contrast, the realization of the peace accord motivated the consolidation of new cattle ranching (Fig. 3B,D). The proliferation of coca farming and illegal cattle ranching coincides with shifting land tenure policies associated with the demobilization of the FARC-EP and the handover of the territory to the GoC. These changes influenced actors' expectations for deriving short-term profits from specific land uses^31^. Market liberalization has historically explained land use changes in Colombian agribusinesses^64^, particularly during the coffee boom in the late 70's. Our current analysis shows that illicit activities in peripheral regions have also responded to the land use liberalization that emerged in the post-conflict period. Nevertheless, the increases in cattle ranching well beyond the agricultural frontier and coca cultivation in protected areas during the last six years represent a new threat to forest conservation in the Amazon.

The temporal expansion of coca farming is cyclical but remains insignificant in terms of area (~ 37.000 ha). Additionally, patterns of forest regeneration are more important than any other conversion associated with coca. Forest regeneration patterns are essential to prioritizing conservation efforts where illegal cattle ranching has not yet consolidated. While coca farming remains spatially concentrated, cattle ranching—the only viable agricultural alternative—is widely distributed across the Amazon and, as we show, is now deeper into Amazon. Explanations for cattle investment are multifaceted. A fundamental explanation for expanding cattle ranching lies in the logic of settler colonialism as immigrants exploit newly available lands in the frontier, propelling short-term economic booms^65^. A second explanation is the historical role that landownership and ranching play in upward social mobility and political power^66^. Ranchers acquire pasture lands to enhance political participation, as well as to guarantee control over territory and resources. While profits and control are underlying elements to explain cattle dynamics, it is the land policies that make the consolidation of cattle ranching possible^8^.

We identify three land policies that accelerate forest loss from coca and cattle in the Colombian Amazon: (1) titling laws requiring agricultural productivity which favor large landowners and consolidation, (2) coca eradication programs that push production deeper into protected areas, and (3) insufficient implementation of peace accord elements to improve local forest governance. Land concentration by ranchers and elites confer disproportionate power over their surrounding local communities^67^. Under diverse legal or illegal mechanisms, the main aim is to acquire control over local governments and institutions that perpetuate land accumulation. Consequently, land policies have commonly privileged large landowners over smallholders. Land policies create legal opportunities for legal titling if landholders could provide evidence of land possession and agricultural land productivity on 75% of total farms^21,39^. The most straightforward way to claim productivity on large landholdings is cattle ranching. However, cattle ranching requires clearing significant forest areas and planting pasture to prevent secondary forest regeneration, neither of which are inexpensive activities^65^. *Forest to cattle* conversion thus requires an initial investment that farmers can only undertake with access to capital. While poor landless farmers also take advantage of this process, it is only temporary. A common economic strategy for landless farmers is clearing forested land, converting it to pasture, and "flipping" it for sale to better capitalized buyers. Well-capitalized landholders then buy farms from smallholders that cannot demonstrate land productivity. After selling lands in informal land markets, with no other local agricultural opportunities, landless farmers move from relatively consolidated areas deeper into the forest. Consequently, any attempt by the government to provide land to landless farmers is quickly repressed by powerful landowners. Taken together, in the peripheral lands of the Amazon, the aim of land acquisition by large landowners is not profiting from cattle ranching but rather securing expectations about the future value of land and speculating through illegal land markets^68,69^. Our results support this explanation, with a gradual linear increase in cattle ranching (Fig. 3B) until the signing of the peace accord in 2016 when both *forest to cattle* and cattle consolidation grew rapidly (Fig. 3D).

In contrast to land policies that accelerate cattle expansion, billions of dollars have been invested to stop coca farming over the last 40 years^70^. However, our findings indicate that coca farming continues to expand. Our time-series analysis reveals that the timing of coca farming adds weight to the claim that eradication programs have only locally attenuated coca cultivation, even while displacing it deeper into PAs and outside the frontier (Fig. 3A,C). Likewise, we show that coca farming persists even after coca substitution programs have implemented agriculture alternatives and—similar to what is observed in Central America—interdiction also displaces narcotrafficking deeper into PAs^71,72^. New land use policies associated with the peace accord such as PNIS, still do not address the land claims and needs of small coca farmers. PNIS provides assistance to farmers outside PAs to establish agricultural projects and for farmers living within PAs, economic incentives for restoration. However, the Colombian Constitution restricts any agriculture activities within PAs, and the restoration itself does not solve the livelihood issue of the families living in PAs. At the end of 2019, only 1% of the *campesino* families had an agricultural project^46^. While the GoC continues focused on eradication policies rather than the recognition of legal titles, the permanence of families within PAs, and long-term goals for improving socio-economic conditions, coca cultivation will likely follow the same spatio-temporal dynamics because it still provides high, steady, short-term economic returns^40,73^.

The GoC began the negotiation process by recognizing that peace was necessary for continued economic growth: by 2010, legally available land within the agricultural frontier was already occupied and consolidated. The next frontier included territory held by FARC-EP. The explosion of illegal cattle ranching that we document should come as no surprise. It was the next logical step for Colombia's economy. The recent conversion patterns tell us about local priorities in the Amazon: coca farming, cattle ranching, and the land markets that support them coincide with local livelihood needs and large landowners' interests. While cattle and coca are not mutually exclusive activities during armed conflict, the narco-cattle ranching link is a common strategy for territorial control and money laundering^9^. Recent efforts by the Colombian land agency established new norms to develop a set of forest reserves that guarantee forest sustainability and low-impact activities with management conditions by the local communities. Additionally, a conceptual change in forest conservation recognizes the role of the community in the management and building strategies for their well-being. Practical examples of success in other armed conflictive regions, such as the Peten in Guatemala, showed promising regional success. Through forest concessions and product diversification, suitable economic returns to community members and the protection of natural resources have been achieved^74^. In Colombia, key aspects of the peace accords have been poorly implemented, but could directly contribute to halting narco-cattle^46^. The agreement includes strengthening governance at the local level through community-based land management, local political participation, and serious alternatives to coca farming. For instance, VisionAmazonia, an initiative to reduce deforestation, has promoted agro-environmental development through several pathways. VisionAmazonia aims to prioritize value chains for sustainable non-timber products, promote ecotourism, and support the conversion of pastureland back to forest (i.e., *rastrojo*). Practical examples in Guaviare are protecting ~ 30.000 ha through forest conservation and ecotourism strategies^61^. In Putumayo, farmers have also begun to plant native timber-yielding varieties to gradually recuperate diverse vegetation, soils, insects, microbial life, and watershed areas^60,75^. While *rastrojo* is viable for the natural recovery of soils and the environment, funding has yet to reach large areas and communities. Instead, remaining rebel groups continue to pressure farmers to farm coca, and the common promotion of cattle expansion as a unique agricultural alternative has posed daily persistent challenges for forest conservation during this post-conflict period. Illicit cattle ranching is responsible for most of the deforestation, and while coca has expanded deeper into PAs, it remains a small fraction of total deforestation. To better capture the individual drivers of forest loss, future work should focus on improving coca farming accuracy because it may be confounded with small subsistence agriculture in some regions. Additional field data at forested sites cleared for pasture as well as forests cleared for a land grab should be collected to support improved discrimination of these distinct drivers that have different ecological and policy implications. To account for the plurality of factors contributing to forest loss dynamics, we recommend blending academic and local knowledge to build a near-real-time remote sensing system to monitor landscape change, create awareness, and expand "satellite activism"^76^ for forest conservation^77^. Detailed land censuses and significant tax payments for larger landholders are also necessary to help stem the risk of illicit land markets appearing in post-conflict territories. This case study provides an applied framework that could be used elsewhere to disentangle illicit land uses, quantify their presence, understand their causes, and develop policies to manage them for the well-being of people and the environment. The availability of frequent Earth Observation data resolves a key challenge of examining the role of historical and institutional processes in the expansion of illicit land activities. This framework could shed light on emerging thorny issues in land system science, including expansion of informal urban peripheries^78,79^, illegal commercial agriculture^80^, illegal lumber harvesting^3^, illegal fishing and aquaculture^81^, and oil exploration leading to deforestation^82^.

## Methods

All available Landsat Collection 1 surface reflectance images from 1984 to 2019 were used in the analysis. The satellite image dataset was then used to produce a time-series of radiometrically consistent, annual composites using the LandTrendr algorithm (e.g., ^83,84^). Reference data for coca, cattle, and forest plots come from official and published sources on an annual basis from 2009 to 2018. Coca patches were partially obtained only within Protected Areas from SIMCI. Coca plots are visually delineated using high-resolution imagery and confirmed by aerial inspection^34^. Cattle and forest plots were obtained through the combination of land cover maps within and outside PAs^30^ and Corine Land Cover (CLC) data^85^. The study area was spatially stratified into ~ 20 km^2^ blocks. Inside each spatial partition, we extracted overlapping 128-by-128 pixel subsets of Landsat imagery and the reference labels. The model dataset consisted of 329,804 examples. More details about the image processing, reference generation, and accuracy assessment are included in the SI.

Coca and cattle ranching lands were classified using the UNet deep learning architecture^86^. UNet resembles a traditional convolutional autoencoder architecture (e.g., Vincent et al.^87^), but has additional "skip-connections" (i.e., feature maps produced by the encoder are concatenated with feature maps produced by the decoder) which improves the accuracy of the segmentation. The encoder network in the UNet architecture was replaced with the MobileNetV3 encoder^88^. Hyperparameters were selected using 25 iterations of a random search over the parameter space using an 80–20%, spatially stratified, train-test split of the modeling dataset^89^. The model's weights were optimized using ADAM^90^. Model validation was conducted using a spatially-stratified fivefold cross-validation procedure^91^. Each model developed during cross validation was applied to the time series of Landsat composited imagery. The five separate classification time series were then merged into a final classified time-series by taking the mode of the five predicted values at each pixel location for each year of the time series. For more details on model development, validation, and inference, see the SI. A website with land transitions maps is available: https://murillop.users.earthengine.app/view/cocacattle.

## Supplementary Information

Supplementary Information.

## Data Availability

The datasets generated and/or analyzed during the current study are not publicly available due to the sensitivity of the population affected including small farmers and the potential harm that could arise from sharing the specific location of illicit land activities. However, the dataset is available from the corresponding author on reasonable request. A website with land transition maps is available: https://murillop.users.earthengine.app/view/cocacattle.
