# Supplementary material for: The post-conflict expansion of coca farming and illicit cattle ranching in Colombia
Source: Sci Rep. 2023 Feb 3;13:1965. doi: 10.1038/s41598-023-28918-0 (PMC9898308; doi:10.1038/s41598-023-28918-0)
Supplement: Supplementary file 1 — Supplementary Information. [file 41598_2023_28918_MOESM1_ESM.docx]

# Supplementary Information for

**The post-conflict expansion of coca farming and illicit cattle ranching in Colombia**

Paulo J. Murillo-Sandoval^1,2, *^, John Kilbride^1^, Elizabeth Tellman^3^, David Wrathall^1^, Jamon Van Den Hoek^1^, Robert E. Kennedy^1^

^1^College of Earth, Ocean and Atmospheric Sciences, Oregon State University, Corvallis, United States

^2^Departamento de Topografía, Facultad de Ciencias del Hábitat, Diseño e Infraestructura, Universidad del Tolima, Ibagué, Colombia

^3^School of Geography, Development, and Environment, University of Arizona, Tucson, Arizona, United States

# *Corresponding author: Paulo J. Murillo-Sandoval

# Email: [pjmurillos@ut.edu.co](mailto:pjmurillos@ut.edu.co)

# Study area

# The study area is defined by the biogeographical limit corresponding to the Colombian Amazon ecosystem. The boundary was obtained from Sinchi Amazonic Institute of Scientific Research (Instituto Amazónico de Investigaciones Científicas). Colombian Amazon covers 42% of the country's territory, it remains forested 75%, with 6% in pasturelands and 1% illegal crops (Sánchez-Cuervo *et al.*, 2012). The northern portions of the study area are hotspots of deforestation due to anthropogenic activity (e.g., establishment of pastures or agriculture, urbanization, etc.). The southern portions of the Colombian Amazon have very low levels of deforestation.

# Framework

# We follow an illicit land transactions framework defined by Tellman et al (2020a) using two pixel-based approaches. First, we draw hypotheses linking the forest-coca-cattle pattern with specific processes. In this case, the distinctive conversions patterns between coca and cattle helps to infer where, how, and why these illicit activities take place. The reconstruction of these changes were based on ethnographic research collected in 2019 with FARC ex-combatants, farmers, and park managers in this region and on previous studies (Arcila, 1989; Espinosa, 2004; UNODC, 2009; Urdaneta, 2017; Gootenberg and Davalos, 2018; Van Dexter and Visseren-Hamakers, 2019). Second, we directly map the spatiotemporal distribution of illicit activities using partially known locations of coca and cattle through a deep learning model.

# Satellite data

# To map the distribution of coca and pasture, we used multispectral satellite imagery acquired by the Landsat program. All surface reflectance Landsat images from the U.S. Geologic Survey’s Collection 1 dataset which intersected our study area and which were acquired between 1984 to 2019 were aggregated for analysis. Imagery acquired by the Landsat Thematic Mapper and Enhanced Thematic Mapper plus sensors were harmonized with data acquired by the Operational Land Imager sensor using the reduced major axis regression coefficients from Roy et al. 2015. Additionally, each image was corrected for topographic effects (Shepherd and Dymond, 2003). An annual time series of wall-to-wall composites was produced using the using the medoid method for the period 1984-2019 (Flood, 2013). The LandTrendr algorithm was used to produce a radiometrically consistent, gap-free time series of annual satellite imagery (Kennedy et al., 2010, Kennedy et al., 2018). For additional information about using LandTrendr to produce a radiometrically consistent, gap-free time series see Kennedy et al. 2015. Finally, to supplement the annual time series of Landsat imagery, we gathered elevation data from the Shuttle Radar Topography Mission digital elevation model (DEM) dataset (Farr *et al.*, 2007). All image processing was conducted using the Google Earth Engine cloud computing platform (Gorelick *et al.*, 2017).

# Reference data

# Coca, cattle and forest plots come from official and published sources on an annual basis from 2009-2018. Coca patches were partially obtained only within Protected Areas from SIMCI. Coca plots are visually delineated using high-resolution imagery and confirmed by aerial inspection (UNODC-SIMCI, 2017). While methodological limitations for coca plots remain and comparison with statistical projections suggests that coca area tends to be underestimated (Uribe, 2019), coca records within PAs are well-documented to be employed as training data. Cattle and forest plots were obtained through the combination of land cover maps within and outside PAs (Murillo-Sandoval *et al.*, 2018); Murillo et al., in review) and Corine Land Cover (CLC) map. Cattle farms size might range between 80 and 390 ha; the remaining forest within them varies between 50-63% leading to smaller areas exclusively dedicated to cattle (UAESPNN, 2015). To improve cattle detection, annual pastures were spatially constrained using CLC, and only patches greater than 25 ha were selected. We further corroborated cattle areas using high-resolution imagery available on Google Earth.

# Training dataset

# We developed a dataset capable of training a semantic segmentation deep learning model using the wall-to-wall time-series of composited satellite imagery and the wall-to-wall reference maps. To develop the dataset, we first spatially partitioned the study area into a 19,230m grid (Fig S1). From each spatial partition, we extracted training examples to populate the modeling dataset. Each training example consists of a 128-by-128-pixel window extracted from of the Landsat imagery and DEM and a spatially coincident 128-by-128-pixel window extracted from the reference map. To maximize the amount of data drawn, we extract overlapping windowed subsets from each spatial partition (Fig S2). Each example was allow to overlap previous example drawn by 96 pixels. Within each partition, we repeated the overlapped extraction for each year we possessed reference data. Given we possessed a census of coca locations in 2008, all possible 128-by-128 examples were drawn from all spatial partitions in 2008. For the other years of the time-series, only examples where the reference label contained coca were included in the modeling dataset. This sampling procedure was found to balance the label error rate and helped to address the imbalance between the classes. In total, 329,804 examples were extracted to form the modeling dataset used in this analysis.

#
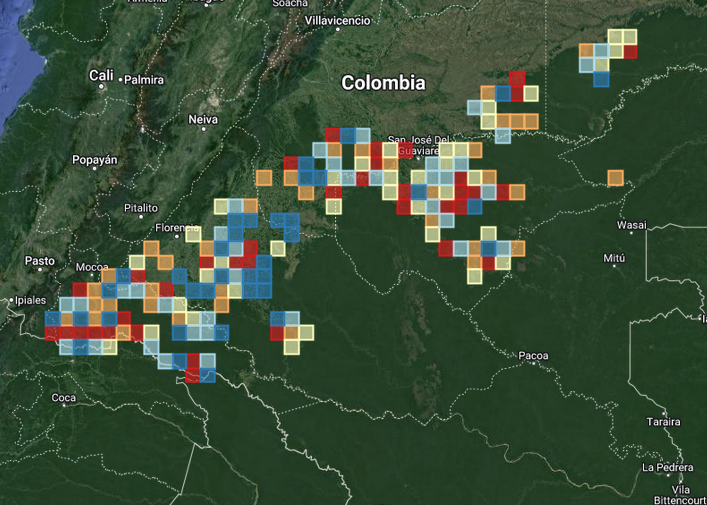


**Fig S1**. The 19,320m spatial partitions used in this analysis to stratify the study area. The colors correspond to the modeling folds used during the spatial cross validation procedure. Red is fold 1, orange is fold 2, yellow is fold 3, light blue is fold 4 and dark blue is fold 5. Map created in QuantumGIS 3.16 (<https://www.qgis.org/en/site/>).


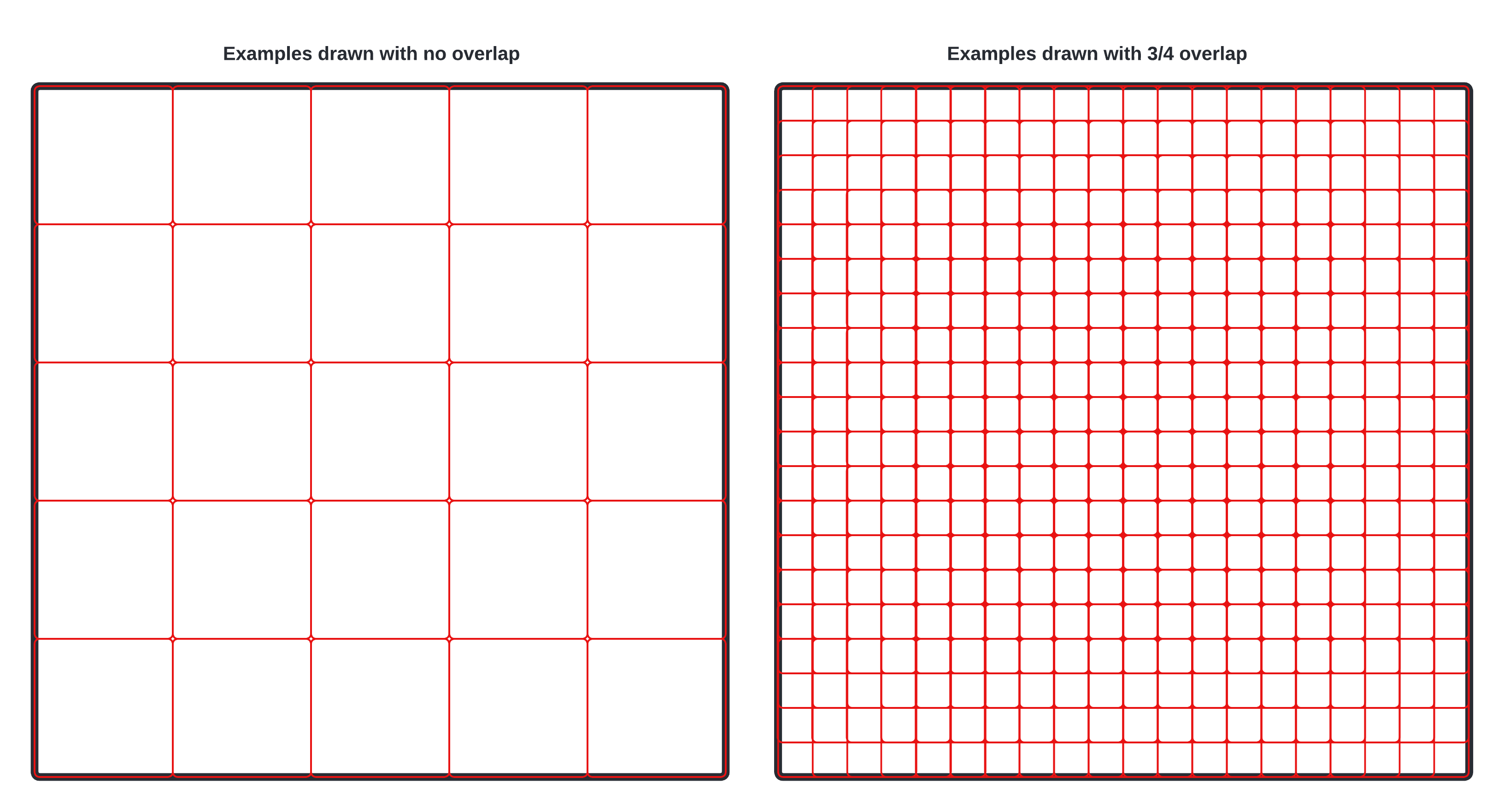


**Fig S2.** A schematic showing how samples (red outlines) were drawn from each spatial partition (black). The right exhibits how 128-by-128 pixel examples could be extracted if each example was prohibited from overlapping. The left-hand side of the figure shows how examples were extracted in this analysis. Note that the squares are the same size on each figure but the squares on the left are overlap previous examples (horizontally and/or vertically) by 96 pixels.

# Model Architecture

# In this analysis, we used the UNet semantic segmentation architecture (Ronneberger, Fischer and Brox, 2015) to classify coca and pasture. The UNet architecture resembles a traditional convolutional autoencoder but incorporates skip-connections which concatenate feature maps produced by various layers in the encoder network with the feature maps produced by layers of the decoder network. These skip-connections allow for the feature maps produced encoder to be re-used in the decoder and significantly improve the quality of the segmentation results. The UNet model used in this analysis replaced the encoder network described in Ronneberger et al. 2015 with a MobileNetV3-style encoder (Howard *et al.*, 2019). MobileNetV3 is a computationally efficient CNN architecture that was developed for mobile computing platforms. The model used in this analysis was implement using the PyTorch deep learning library (Paszke *et al.*, 2019). During preliminary testing, we note that the deeper encoder architectures we evaluated (e.g., ResNet50, ResNeSt50, ResNeSt269) did not meaningfully improve the neural networks generalization capabilities to the testing set (He *et al.*, 2015; Zhang *et al.*, 2020)

# Model training and validation

# Hyperparameters were selected using 25 iterations of a random search for with an 80/20 split of the data (Bergstra and Bengio, 2012). A learning rate of 0.0275 and a batch size of 128 yielded the best performance and were used when training all subsequent models. Model validation was performed using a spatially stratified, 5-fold cross-validation procedure (Fig S1; (Roberts *et al.*, 2017). During each iteration of the cross-validation procedure, the examples from 80% of the spatial partitions were allocated to the training set and the examples from the remaining partitions were allocated to the testing set. Tanimoto loss, a differentiable form of intersection over union (IoU) score, was used as cost function. Tanimoto loss is defined as

$T\left( p,l \right)=\frac{\sum p_{i}l_{i}}{\sum\left( p_{i}^{2}l_{i}^{2} \right)-\sum\left( p_{i}l_{i} \right)}$

# ...where *p* is a value between 0 and 1 and gives the probability for each class at the *i*^th^ pixel location and *l* is a binary vector indicating which class is present at *i*^th^ pixel location (Diakogiannis *et al.*, 2020). During training, we tracked the accuracy and the IoU score. For each metric, we computed each score using an unweighted average which results in a more conservative estimate of accuracy as we are weighting each class equally. We report the mean value of the cross-validation and the confidence envelope (+/- 2 standard deviations) for each metric. Model parameters were optimized using ADAM (Kingma and Ba, 2014). Early stopping, a technique where the test set loss is monitored during training to monitor for over-fitting (i.e., decreased generalization accuracy), was used during the cross validation procedure. The PyTorch-Lightning library was used to facilitate distributed processing across two Nvidia RTX 2080 Ti graphical processing units (GPUs) (Falcon *et al.*, 2020).

# The models developed during the cross-validation procedure had a training accuracy of 79.7% (+/- 0.006) and a IoU score of 0.72 (+/- 0.006) (Fig S3). The models developed in this analysis had a validation accuracy of 0.72 % (+/- 0.007) and an IoU score of 0.71 (+/- 0.006) (Fig S4). The background class had a validation IoU score of 0.92 (+/- 0.013). The pasture class had a validation IoU score of 0.92 (+/- 0.014). The coca class had a validation IoU score of 0.62 (+/- 0.04).


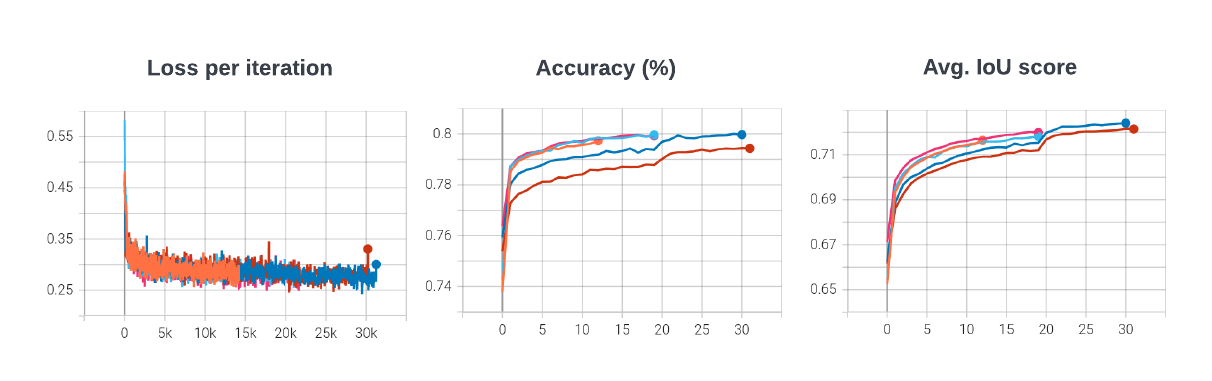
**Fig S3.** Training set statistics aggregated during the cross validation procedure. The first panel shows the loss computed during each iteration of training (Jaccard loss). The second panel shows the average accuracy of the model (across all classes), with respect to the training set, after each epoch of training. The third panel shows the average Intersection over Union (IoU) score of the model (averaged across all classes), with respect to the training set, after each epoch of training.


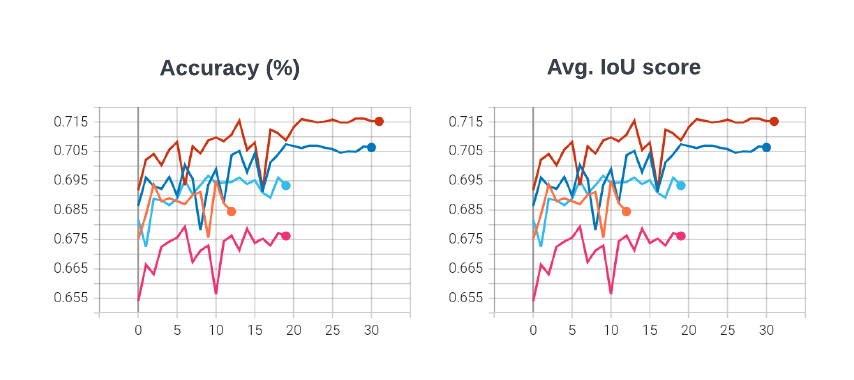


**Fig S4.** Test set statistics aggregated during the cross validation procedure. The first panel shows the average accuracy of the model (across all classes), with respect to the test set, after each epoch of training. The third panel shows the average Intersection over Union (IoU) score of the model (averaged across all classes), with respect to the test set, after each epoch of training. Note, although the two plots are similar, the actual values between the curves differ by small amounts.

# Inference procedure

# Each of the 5 models developed during cross-validation procedure were applied to the entire time series of composited Landsat imagery. Fully convolutional neural networks, such as UNet, can be applied to images with arbitrary dimensions. However, classifying the entire study region at once would require very large amounts of GPU memory. Instead, each image in the time series was first broken into smaller 128-by-128 subsets which were individually classified and then reassembled to obtain a land cover map with wall-to-wall coverage. To avoid edge effects during inference, only the center 64-by-64 pixel subset of each 128-by-128 subset was used to create each classified map. Applying each of the 5 models to the time series of Landsat composites produced 5 classified time-series. For each year of classifications, the mode of the five maps was computed at each pixel location which yielded the final classified time series.

# Area estimation

# After classified time series for land uses classes an independent accuracy assessment was created. Initially, a random stratified procedure was elaborated to evaluate areas estimation. We follow (Olofsson *et al.*, 2014) approach to evaluate the accuracy of nine change classes between *forest-cattle-coca*. The most common transition is *forest to cattle* from which we determined the sample size using a standard error of 0.5%. We anticipated an accuracy for *forest to cattle* equals to 0.8 and a proportion of this class in the rest of change classes of 1%. Using this information, we found a sample size of 550 samples. We allocated 300 samples for *stable forest*, 50 for *forest to cattle* and 30 for the rest of the classes (total allocation of 560 samples). The analysis was built for the change period 1987-2019. We employed Collect Earth to link samples with reference information from Landsat subsets, NDFI time-series and Planet data. A summary of accuracy and area estimation from each change class is presented in Table S1.

# Table S1. Area estimates, confidence intervals and accuracies for the nine change classes using the map change 1987-2019.

|  | Class | Area Estimated (ha) | CI (95%) | MoE | Area Mapped (ha) | Map Bias | Producer's | User's |
| --- | --- | --- | --- | --- | --- | --- | --- | --- |
| 1 | *forest to coca* | 30632.81 | 125.569 | 0.409916687 | 30442.47862 | 190.331377 | 0.994 | 1 |
| 2 | *forest to cattle* | 1977252.765 | 163252.335 | 8.256523288 | 2149128.679 | -171875.9139 | 1 | 0.92 |
| 3 | *stable natural* | 44498299.7 | 142909.139 | 0.321156404 | 44369351.97 | 128947.721 | 0.997 | 1 |
| 4 | *cattle to coca* | 435.044 | 148.113 | 34.04552183 | 815.7072563 | -380.6632563 | 1 | 0.533 |
| 5 | *stable cattle* | 993255.766 | 106975.602 | 10.77019693 | 973936.5551 | 19319.2109 | 0.948 | 0.967 |
| 6 | *cattle to forest* | 278925.03 | 65927.384 | 23.63623802 | 255234.4763 | 23690.55372 | 0.884 | 0.966 |
| 7 | *stable coca* | 174.439 | 74.057 | 42.45438233 | 120.0589642 | 54.38003575 | 0.688 | 1 |
| 8 | *coca to cattle* | 6091.217 | 737.621 | 12.10958336 | 6737.807134 | -646.5901343 | 0.996 | 0.9 |
| 9 | *coca to forest* | 11117.007 | 737.621 | 6.635068234 | 10416.03618 | 700.9708185 | 0.937 | 1 |
|  |  |  |  |  |  |  |  |  |


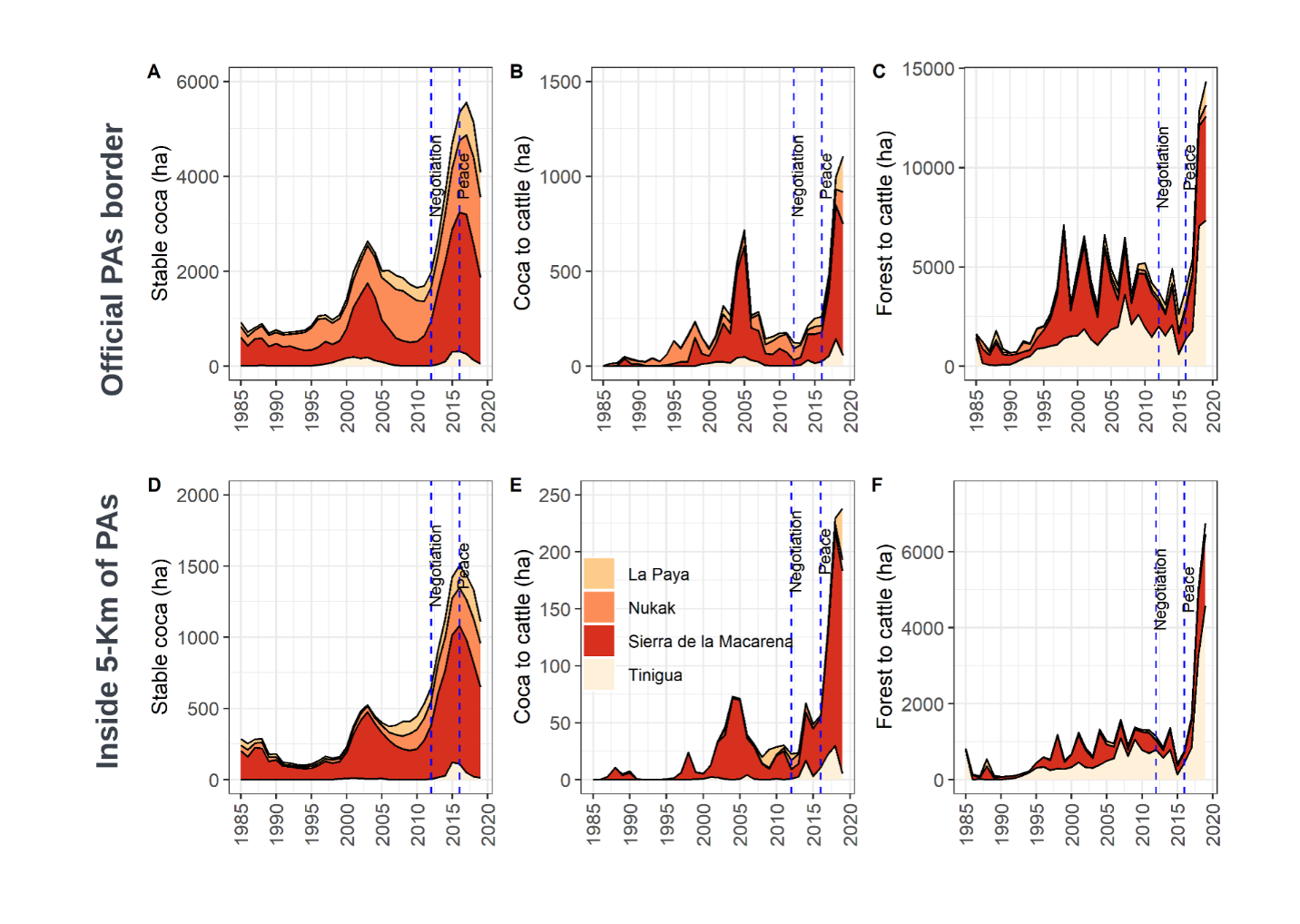


Fig S5. Patterns of three common *forest-coca-cattle* pathways in Protected Areas: Stable coca. Coca to cattle and Forest to cattle. First row shows the mapped areas within the official border of Pas. Second row indicates the mapped areas within the core of the PAs, in other words inside official border by 5km buffer.

**Table S2**. Mapped area (ha) for all transitions forest-coca-cattle outside of agricultural frontier.

| **year** | **cattle to coca** | **cattle to forest** | **forest to cattle** | **stable cattle** | **coca to cattle** | **coca to forest** | **forest to coca** | **stable coca** | **stable forest** |
| --- | --- | --- | --- | --- | --- | --- | --- | --- | --- |
| **1985** | 43.51 | 22970.75 | 15444.96 | 180416.48 | 7.42 | 106.82 | 347.50 | 6497.33 | 43655326.61 |
| **1986** | 185.58 | 28437.13 | 23781.85 | 167246.14 | 126.22 | 1247.48 | 2160.10 | 5514.64 | 43652462.24 |
| **1987** | 654.53 | 45186.64 | 21545.89 | 145313.04 | 133.28 | 1784.77 | 2717.31 | 5942.28 | 43657883.65 |
| **1988** | 480.78 | 22993.72 | 19044.85 | 143517.71 | 305.84 | 1881.94 | 2009.84 | 7126.34 | 43683800.35 |
| **1989** | 271.87 | 16234.75 | 27205.34 | 146361.78 | 331.38 | 2991.50 | 1947.97 | 6294.09 | 43679522.71 |
| **1990** | 213.15 | 14986.34 | 27355.39 | 158699.01 | 224.70 | 1996.76 | 1247.35 | 6292.47 | 43670146.21 |
| **1991** | 140.28 | 22842.37 | 21738.24 | 163296.46 | 200.37 | 2170.90 | 1168.01 | 5381.70 | 43664223.07 |
| **1992** | 105.59 | 10798.57 | 17091.92 | 174330.91 | 243.20 | 1393.51 | 1539.69 | 5053.28 | 43670604.73 |
| **1993** | 221.49 | 10029.06 | 18345.96 | 181415.48 | 135.69 | 1115.13 | 2044.21 | 5447.74 | 43662406.64 |
| **1994** | 262.02 | 14058.22 | 17118.74 | 185576.89 | 195.91 | 1234.96 | 3966.86 | 6282.57 | 43652465.22 |
| **1995** | 254.61 | 11416.82 | 15592.08 | 191220.11 | 342.82 | 1646.65 | 3882.39 | 8521.99 | 43648283.92 |
| **1996** | 220.69 | 10468.90 | 17089.51 | 196465.42 | 456.14 | 1991.17 | 2577.82 | 10211.69 | 43641680.05 |
| **1997** | 281.19 | 15090.74 | 31314.18 | 198639.14 | 829.09 | 3284.16 | 2502.99 | 8896.95 | 43620322.95 |
| **1998** | 490.81 | 16033.56 | 38481.65 | 214258.03 | 791.51 | 2247.87 | 3505.69 | 8641.75 | 43596710.51 |
| **1999** | 583.92 | 23029.66 | 25976.24 | 229917.62 | 767.24 | 2117.42 | 3804.77 | 9753.58 | 43585210.93 |
| **2000** | 724.39 | 16146.75 | 36536.77 | 239789.97 | 707.58 | 1825.79 | 4817.52 | 11608.90 | 43569003.72 |
| **2001** | 667.21 | 14293.64 | 41930.12 | 262073.47 | 1125.23 | 2476.95 | 5379.02 | 13548.63 | 43539667.11 |
| **2002** | 706.73 | 20700.62 | 38361.39 | 283721.48 | 2086.51 | 3944.25 | 5012.19 | 13564.10 | 43513064.12 |
| **2003** | 998.33 | 20116.82 | 37228.54 | 303054.23 | 2340.02 | 3822.19 | 3197.68 | 13120.81 | 43497282.78 |
| **2004** | 765.74 | 27315.78 | 38661.69 | 314541.26 | 2028.72 | 3784.74 | 3709.80 | 11503.36 | 43478850.30 |
| **2005** | 317.87 | 24350.31 | 36893.68 | 330563.49 | 2376.81 | 3965.37 | 2580.11 | 9636.72 | 43470477.04 |
| **2006** | 373.39 | 24705.74 | 44536.05 | 344754.85 | 1055.22 | 2753.65 | 2478.99 | 8725.83 | 43451777.67 |
| **2007** | 287.43 | 27003.19 | 57464.01 | 363055.50 | 1063.28 | 2630.33 | 1942.86 | 7884.60 | 43419830.20 |
| **2008** | 414.81 | 36438.60 | 36622.64 | 384729.37 | 554.46 | 2371.60 | 1895.08 | 7188.83 | 43410945.99 |
| **2009** | 219.09 | 28229.31 | 48633.71 | 393458.08 | 1009.71 | 2451.22 | 1813.40 | 6037.79 | 43399309.08 |
| **2010** | 209.68 | 23560.04 | 69808.32 | 419331.78 | 721.97 | 1878.58 | 1958.48 | 5469.73 | 43358222.81 |
| **2011** | 348.05 | 39714.75 | 58454.98 | 449799.26 | 724.97 | 2145.15 | 1751.15 | 4767.78 | 43323455.30 |
| **2012** | 639.25 | 38706.05 | 61060.16 | 469633.90 | 419.13 | 1536.79 | 2265.29 | 4911.06 | 43301989.74 |
| **2013** | 1035.28 | 36747.70 | 62639.44 | 493330.21 | 411.78 | 1365.75 | 3968.88 | 6038.08 | 43275624.26 |
| **2014** | 878.28 | 32327.63 | 69769.95 | 523175.52 | 643.20 | 1507.79 | 5258.10 | 8891.25 | 43238709.67 |
| **2015** | 1019.25 | 35025.53 | 60684.18 | 557543.90 | 891.39 | 2222.37 | 5146.32 | 11913.87 | 43206714.59 |
| **2016** | 771.61 | 28756.31 | 72999.77 | 589591.54 | 1338.92 | 2450.91 | 4825.46 | 14289.61 | 43166137.25 |
| **2017** | 636.67 | 34216.77 | 96157.57 | 629076.79 | 1850.19 | 3294.96 | 5146.83 | 14741.53 | 43096040.07 |
| **2018** | 498.98 | 29211.50 | 147199.70 | 697374.07 | 2918.89 | 3879.33 | 2131.99 | 13726.82 | 42984220.11 |
| **2019** | 1285.87 | 32440.66 | 189503.10 | 813766.13 | 2406.40 | 2610.14 | 10811.73 | 11341.24 | 42816996.12 |

**Table S3**. Mapped area (ha) for all transitions forest-coca-cattle inside of agricultural frontier.

| **year** | **cattle to coca** | **cattle to forest** | **forest to cattle** | **stable cattle** | **coca to cattle** | **coca to forest** | **forest to coca** | **stable coca** | **stable forest** |
| --- | --- | --- | --- | --- | --- | --- | --- | --- | --- |
| **1985** | 8.04 | 12354.06 | 48082.09 | 905358.35 | 8.49 | 45.84 | 66.85 | 5442.23 | 2942285.09 |
| **1986** | 200.34 | 27939.94 | 95319.72 | 925308.65 | 255.85 | 814.42 | 1966.95 | 4446.85 | 2857398.31 |
| **1987** | 986.24 | 49300.93 | 92026.57 | 970597.06 | 289.79 | 1320.21 | 1981.97 | 5004.14 | 2792144.14 |
| **1988** | 517.54 | 45803.20 | 73150.10 | 1016592.68 | 427.09 | 1174.42 | 1510.98 | 6370.84 | 2768104.19 |
| **1989** | 315.32 | 26656.59 | 119209.78 | 1063197.96 | 627.45 | 1935.30 | 1985.91 | 5836.60 | 2693886.12 |
| **1990** | 262.37 | 32542.37 | 102105.26 | 1150230.45 | 510.01 | 1624.25 | 1167.70 | 6003.57 | 2619205.06 |
| **1991** | 176.62 | 35474.19 | 102096.33 | 1217194.91 | 576.87 | 1799.76 | 1173.87 | 5057.00 | 2550101.48 |
| **1992** | 123.39 | 17147.56 | 87149.97 | 1302597.16 | 418.04 | 1321.09 | 1562.94 | 4668.37 | 2498662.52 |
| **1993** | 262.26 | 13109.34 | 78408.54 | 1376793.57 | 301.94 | 1074.98 | 1703.85 | 4977.78 | 2437018.78 |
| **1994** | 360.05 | 17473.24 | 74373.19 | 1437670.77 | 325.38 | 1226.99 | 2788.86 | 5391.52 | 2374041.05 |
| **1995** | 239.36 | 15002.31 | 66304.20 | 1497127.66 | 478.99 | 1465.47 | 2359.66 | 6595.97 | 2324077.42 |
| **1996** | 305.79 | 14154.52 | 61885.67 | 1549450.53 | 679.54 | 1602.83 | 1943.58 | 6912.62 | 2276715.95 |
| **1997** | 626.05 | 19280.82 | 96646.59 | 1592108.87 | 981.49 | 1920.13 | 2350.45 | 6260.37 | 2193476.25 |
| **1998** | 721.61 | 23082.28 | 98784.55 | 1665933.06 | 1040.32 | 1279.71 | 2817.95 | 6916.83 | 2113074.72 |
| **1999** | 1388.07 | 29309.75 | 74125.44 | 1735060.11 | 879.32 | 1185.93 | 3625.09 | 8391.14 | 2059686.19 |
| **2000** | 959.75 | 24121.28 | 90378.12 | 1784983.84 | 1529.80 | 1337.75 | 3681.82 | 10536.73 | 1996121.94 |
| **2001** | 887.96 | 22347.93 | 88608.34 | 1853655.87 | 1827.09 | 1566.14 | 3639.87 | 11785.07 | 1929332.77 |
| **2002** | 786.37 | 29303.04 | 77553.21 | 1914001.90 | 2717.71 | 2417.60 | 3209.88 | 11177.59 | 1872483.75 |
| **2003** | 796.88 | 30319.21 | 73110.33 | 1963156.73 | 3051.33 | 2493.83 | 2009.33 | 9628.68 | 1829084.72 |
| **2004** | 481.15 | 34824.45 | 75793.67 | 2004012.79 | 2432.83 | 2174.52 | 1626.44 | 7827.53 | 1784477.65 |
| **2005** | 366.11 | 33262.33 | 70580.10 | 2048610.85 | 1869.05 | 1821.91 | 1171.79 | 6244.17 | 1749724.72 |
| **2006** | 338.18 | 36493.00 | 75715.25 | 2084228.83 | 998.48 | 1393.68 | 1106.75 | 5389.92 | 1707986.95 |
| **2007** | 261.76 | 35702.72 | 82097.82 | 2124978.07 | 865.56 | 1337.43 | 757.30 | 4631.86 | 1663018.51 |
| **2008** | 263.86 | 47713.59 | 54584.79 | 2159964.01 | 593.34 | 1168.96 | 640.07 | 3888.62 | 1644833.79 |
| **2009** | 209.05 | 40290.41 | 58790.36 | 2174642.68 | 667.47 | 1159.76 | 483.44 | 2965.33 | 1634442.53 |
| **2010** | 197.61 | 37168.88 | 59257.95 | 2196734.02 | 567.28 | 756.81 | 399.99 | 2333.74 | 1616234.76 |
| **2011** | 221.38 | 51183.82 | 34851.47 | 2205154.05 | 608.35 | 557.98 | 314.26 | 1765.00 | 1618994.71 |
| **2012** | 360.44 | 50772.56 | 31552.81 | 2189480.87 | 258.97 | 334.27 | 460.66 | 1707.40 | 1638723.05 |
| **2013** | 778.22 | 51946.23 | 26233.29 | 2168568.21 | 259.03 | 288.40 | 839.28 | 1981.06 | 1662757.32 |
| **2014** | 728.43 | 48572.03 | 24426.61 | 2145760.07 | 273.09 | 248.45 | 924.54 | 3077.01 | 1689640.81 |
| **2015** | 827.57 | 44177.81 | 24307.92 | 2125454.38 | 418.36 | 325.75 | 1311.83 | 3985.87 | 1712841.53 |
| **2016** | 664.63 | 41650.98 | 25497.86 | 2107865.06 | 619.91 | 461.11 | 1133.27 | 5044.25 | 1730713.97 |
| **2017** | 590.38 | 41812.73 | 27459.44 | 2091579.72 | 815.48 | 571.50 | 960.17 | 5455.17 | 1744406.44 |
| **2018** | 454.13 | 40456.52 | 36201.96 | 2078943.99 | 1033.48 | 772.85 | 540.72 | 5199.39 | 1750047.99 |
| **2019** | 1121.13 | 41268.30 | 49130.99 | 2073790.00 | 1130.09 | 618.84 | 2378.16 | 4445.30 | 1739768.22 |

**References**

Arcila, O. H. (1989) ‘Coca, guerrilla, colonización y narcotráfico en la Macarena’, *Revista de la Universidad Nacional*, (21), pp. 75–80.

Bergstra, J. and Bengio, Y. (2012) ‘Random Search for Hyper-Parameter Optimization’, *J. Mach. Learn. Res.*, 13(null), pp. 281–305.

Van Dexter, K. and Visseren-Hamakers, I. (2019) ‘Forests in the Time of Violence’, *Journal of Land Use Science*, 1(1), p. 16. doi: 10.1300/j091v16n03_03.

Diakogiannis, F. I. *et al.* (2020) ‘ResUNet-a: A deep learning framework for semantic segmentation of remotely sensed data’, *ISPRS Journal of Photogrammetry and Remote Sensing*, 162, pp. 94–114. doi: https://doi.org/10.1016/j.isprsjprs.2020.01.013.

Espinosa, N. (2004) ‘" A la otra orilla del rio " La relacion de los campesinos y la guerrilla en la Macarena’. doi: 10.13140/RG.2.1.4864.3048.

Falcon, W. *et al.* (2020) ‘PyTorchLightning/pytorch-lightning: 0.7.2rc3 - Test release before 0.7.2’. Zenodo. doi: 10.5281/zenodo.3743165.

Farr, T. G. *et al.* (2007) ‘The Shuttle Radar Topography Mission’, *Reviews of Geophysics*, 45(2). doi: 10.1029/2005RG000183.

Flood, N. (2013) ‘Seasonal composite landsat TM/ETM+ Images using the medoid (a multi-dimensional median)’, *Remote Sensing*, 5(12), pp. 6481–6500. doi: 10.3390/rs5126481.

Gootenberg, P. and Davalos, L. M. (2018) *The Origins of Cocaine*. Edited by P. Gootenberg and L. M. Davalos. Routledge Taylor Francis Group.

Gorelick, N. *et al.* (2017) ‘Google Earth Engine: Planetary-scale geospatial analysis for everyone’, *Remote Sensing of Environment*, 202, pp. 18–27. doi: 10.1016/j.rse.2017.06.031.

He, K. *et al.* (2015) ‘Deep Residual Learning for Image Recognition’, *CoRR*, abs/1512.0. Available at: http://arxiv.org/abs/1512.03385.

Howard, A. *et al.* (2019) ‘Searching for MobileNetV3’, in *2019 IEEE/CVF International Conference on Computer Vision (ICCV)*, pp. 1314–1324. doi: 10.1109/ICCV.2019.00140.

Kennedy, R. E., Yang, Z. and Cohen, W. B. (2010) ‘Detecting trends in forest disturbance and recovery using yearly Landsat time series: 1. LandTrendr - Temporal segmentation algorithms’, *Remote Sensing of Environment*, 114(12), pp. 2897–2910. doi: 10.1016/j.rse.2010.07.008.

Kingma, D. and Ba, J. (2014) ‘Adam: A Method for Stochastic Optimization’, *International Conference on Learning Representations*.

Murillo-Sandoval, P. J. *et al.* (2018) ‘Detecting and attributing drivers of forest disturbance in the Colombian andes using landsat time-series’, *Forests*, 9(5), pp. 1–16. doi: 10.3390/f9050269.

Olofsson, P. *et al.* (2014) ‘Good practices for estimating area and assessing accuracy of land change’, *Remote Sensing of Environment*, 148, pp. 42–57. doi: 10.1016/j.rse.2014.02.015.

Paszke, A. *et al.* (2019) ‘PyTorch: An Imperative Style, High-Performance Deep Learning Library’.

Roberts, D. R. *et al.* (2017) ‘Cross-validation strategies for data with temporal, spatial, hierarchical, or phylogenetic structure’, *Ecography*, 40(8), pp. 913–929. doi: https://doi.org/10.1111/ecog.02881.

Ronneberger, O., Fischer, P. and Brox, T. (2015) ‘U-Net: Convolutional Networks for Biomedical Image Segmentation’, in Navab, N. et al. (eds) *Medical Image Computing and Computer-Assisted Intervention -- MICCAI 2015*. Cham: Springer International Publishing, pp. 234–241.

Sánchez-Cuervo, A. M. *et al.* (2012) ‘Land Cover Change in Colombia: Surprising Forest Recovery Trends between 2001 and 2010’, *PLoS ONE*, 7(8), p. e43943. doi: 10.1371/journal.pone.0043943.

Shepherd, J. D. and Dymond, J. R. (2003) ‘Correcting satellite imagery for the variance of reflectance and illumination with topography’, *International Journal of Remote Sensing*, 24(17), pp. 3503–3514. doi: 10.1080/01431160210154029.

Tellman, B. *et al.* (2020) ‘Understanding the role of illicit transactions in land-change dynamics’, *Nature Sustainability*. doi: 10.1038/s41893-019-0457-1.

UAESPNN (2015) *Convenio de Asociación Tripartita P.E. GDE.1.4.7.1.14.022 Suscrito entre Parques Nacionales Naturales, Cormacarena y Patrimonio Natural Fondo para la Diversidad y Áreas Protegidas. UAESPNN–Dirección Territorial Costa Orinoquia: Bogota, Colombia; p. 118.*

UNODC-SIMCI (2017) *Monitoreo de territorios afectados por cultivos ilícitos 2017*. Bogota, Colombia. Available at: https://www.unodc.org/documents/crop-monitoring/Colombia/Colombia_Monitoreo_territorios_afectados_cultivos_ilicitos_2017_Resumen.pdf.

UNODC (2009) *Transformacion socioeconomica y biofisica asociadas con cultivos ilicitos en la region sur del Meta-Guaviare 1990-2009*.

Urdaneta, J. S. (2017) *Justicias bastardas: estudio sobre la administración de justicia de la guerrilla de las FARC en el suroriente colombiano*. La Plata.

Uribe, S. (2019) ‘¿Quién tiene la razón en las cifras sobre coca, la ONU o la Casa Blanca?’, *Razon Publica*. Available at: https://razonpublica.com/quien-tiene-la-razon-en-las-cifras-sobre-coca-la-onu-o-la-casa-blanca/.

Zhang, H. *et al.* (2020) ‘ResNeSt: Split-Attention Networks’, *ArXiv*, abs/2004.0.
